# Dr. Ira P. Smith

**Published:** 1905-07

**Authors:** 


Dr. Ira P. Smith, of Bath, N. Y., died at his home May 26, 1905, aged 69 years. He graduated from Albany medical college in 1859, and served as a regimental surgeon during the civil war.

